# The Autophagy-Related Gene *Aolatg4* Regulates Hyphal Growth, Sporulation, Autophagosome Formation, and Pathogenicity in *Arthrobotrys oligospora*

**DOI:** 10.3389/fmicb.2020.592524

**Published:** 2020-11-16

**Authors:** Duanxu Zhou, Meihua Xie, Na Bai, Le Yang, Ke-Qin Zhang, Jinkui Yang

**Affiliations:** State Key Laboratory for Conservation and Utilization of Bio-Resources and Key Laboratory for Microbial Resources of the Ministry of Education, School of Life Sciences, Yunnan University, Kunming, China

**Keywords:** Arthrobotrys oligospora, autophagy-related gene Aolatg4, conidiation, trap formation, autophagosome, stress resistance

## Abstract

Autophagy plays an important role in cell growth and development. The autophagy-related gene *atg4* encodes a cysteine protease, which can cleave the carboxyl terminus of Atg8, thus plays a role in autophagosome formation in yeast and filamentous fungi. *Arthrobotrys oligospora* is well known for producing special trapping-devices (traps) and capturing nematodes. In this study, two Δ*Aolatg4* mutants were generated using targeted gene replacement and were used to investigate the biological functions of autophagy in *A. oligospora*. Autophagic process was observed using the AoAtg8-GFP fusion protein. The mutants showed a defective in hyphal growth and sporulation and were sensitive to chemical stressors, including menadione and Congo red. The spore yield of the *ΔAolatg4* mutants was decreased by 88.5% compared to the wild type (WT), and the transcript levels of six sporulation-related genes, such as *abaA*, *fluG*, *brlA*, and *wetA*, were significantly downregulated during the conidiation stage. Deletion of *Aolatg4* also affected the cell nuclei and mycelial septal development in *A. oligospora*. Importantly, autophagosome formation and the autophagic process were impaired in the Δ*Aolatg4* mutant. Moreover, the Δ*Aolatg4* mutant lost its ability to form mature traps. Our results provide novel insights into the roles of autophagy in *A. oligospora*.

## Introduction

Autophagy is a conserved intracellular recycling process in all eukaryotic cells, in which autophagosomes are delivered to lysosomes or vacuoles for degradation (Reggiori and Klionsky, 2002; Nakatogawa et al., 2012; Ryter et al., 2013). The autophagic process is governed by autophagy-related (Atg) proteins, which orchestrate the different steps of autophagy, and about 19 Atg proteins are necessary for autophagosome formation (Farré and Subramani, 2016). Previous studies have revealed that the ubiquitin-like protein Atg8 localizes to the autophagosome and autophagy-related structures, thus it can be used a reliable marker for autophagy (Hirata et al., 2017). Atg4 is a cysteine protease, which plays a role in the Atg8 conjugation system of autophagy (Ichimura et al., 2000; Kirisako et al., 2000; Maruyama and Noda, 2018). In *Saccharomyces cerevisiae*, Atg8 is C-terminally conjugated to the phospholipid phosphatidylethanolamine (PE) to generate Atg8-PE, and Atg4 cleaves the amide bond of Atg8-PE to release the protein from PE in membranes (Yoshimoto et al., 2004; Nakatogawa et al., 2007, 2012). Therefore, Atg4 plays a vital role in autophagy by cleaving the Atg8-PE, which will help to recycle Atg8 for the next round of the conjugation reaction (Nakatogawa et al., 2007, 2012), and promote the elongation step of the isolation membrane directly (Hirata et al., 2017).

Recently, increasing knowledge of *atg* genes has been acquired from several filamentous fungi (Ying and Feng, 2019). Previous studies showed that Atg proteins play important roles in appressorium formation and virulence in the plant pathogenic fungi, such as *Magnaporthe oryzae*. For example, deletion of *Moatg8* prevented starvation-induced autophagy, appressorium development, and conidium death in *M. oryzae* (Veneault-Fourrey et al., 2006; Liu et al., 2010). Subsequently, deletion of three *atg* genes (*Mgatg1*, *Mgatg5*, and *Mgatg9*), which are required for autophagy, blocked plant infection in *M. oryzae* (Liu et al., 2007, 2010; Lu et al., 2009; Yin et al., 2019), and deletion of *Moatg4* resulted a significant defective in hyphal growth, conidiation, and appressorium formation in *M. oryzae* (Liu et al., 2010, 2012). Moreover, disruption of *Bbatg1* and *Bbatg8* impaired conidial germination and virulence in entomopathogenic fungus *Beauveria bassiana* (Ying et al., 2016). Recently, deletion of *Bbatg11* caused a significantly defective in conidial germination, stress response, and virulence in *B. bassiana* (Ding et al., 2018). Therefore, *atg* genes play important roles in fungal growth, development, and differentiation.

Nematode-trapping (NT) fungi are natural enemies of nematodes since they may attack live nematodes and utilize them as a source of nutrients (Dijksterhuis et al., 1994). *Arthrobotrys oligospora* is a typical species of the NT fungi that can develop adhesive networks (traps) for nematode predation (Yang et al., 2011). In previous study, a knockout mutant of the gene *Aoatg8* was constructed in *A. oligospora* and found that *Aoatg8* is required for conidiation and trap formation (Chen et al., 2013). Similar to *S. cerevisiae*, *M. oryzae*, and *B. bassiana*, most *atg* genes, involved in macroautophagy and selective autophagy, are also conserved in *A. oligospora* (Ying and Feng, 2019). However, little is known about the functions of *atg* genes in *A. oligospora* and other NT fungi. In this study, we elucidated the effect of gene *Aolatg4* on vegetative growth, spore production, and pathogenicity in *A. oligospora* by comparing the biochemical and phenotypical traits between the wild type (WT) strain and Δ*Aolatg4* mutants. Moreover, we observed autophagosome formation and autophagic process in the WT and Δ*Aolatg4* mutants using microscopic analysis of GFP-Atg8. Our results suggest that *Aolatg4* plays important roles in autophagic process, and also regulates hyphal growth, conidiation, stress response, and trap formation in *A. oligospora*.

## Materials and Methods

### Strains and Culture Conditions

The fungus *A. oligospora* (strain no. ATCC 24927) and its derived strains were cultivated on potato dextrose agar (PDA), tryptone glucose (TG), and Corn-maizena Yeast extract (CMY) media as described previously (Yang et al., 2018). The fungal strains were incubated on CM and MM-N media for nutrient stress induced by nitrogen starvation (Talbot et al., 1993). *Saccharomyces cerevisiae* FY834 (strain no. ATCC 90845) was inoculated on SC-Ura medium for screening the recombinational clones (Park et al., 2011). *Caenorhabditis elegans* (strain N2) was incubated on oatmeal medium at 26°C for inducing the trap formation of *A. oligospora* and bioassay.

### Sequence and Phylogenetic Analyses of *AolAtg4*

The autophagy gene *Aolatg4* was retrieved from *A. oligospora* according to the orthologous gene *atg4* in *S. cerevisiae*, and the corresponding protein AolAtg4 (AOL_s00083g501) was identified. The conserved domains of AolAtg4 were predicted using InterProScan.^1^ Similarities of orthologous Atg4 proteins from different fungi were predicted using the DNAman software package (Lynnon Biosoft, San Ramon, United States; Ma et al., 2020). A neighbor-joining tree for Atg4 from various fungi was constructed using the Mega 7.0 software (Kumar et al., 2016).

### Construction of *Aolatg4* Gene Deletion in *A. oligospora*

Deletion of the *Aolatg4* gene was performed using homologous recombination, as described previously (Tunlid et al., 1999; Chen et al., 2013; Zhen et al., 2019). The upstream (2,150 bp) and downstream (2,098 bp) sequences corresponding to the *Aolatg4* ORF (5' and 3' flanking regions) in *A. oligospora* were amplified using paired primers (Supplementary Table S1). Subsequently, the *hph* cassette was amplified using primers Hph-f and Hph-r (Supplementary Table S1), and the plasmid pSCN44 was used as the template (Bernhards and Pöggeler, 2011). Finally, three PCR fragments and a linearized pRS426 vector were co-transformed into yeast strain FY834 *via* electroporation (Bernhards and Pöggeler, 2011; Park et al., 2011). The complete fragment for gene disruption was amplified from the recombinant plasmid pRS426-ATG4-hph using primers AolAtg4-5f and AolAtg4-3r (Supplementary Table S1), then it was transformed into *A. oligospora* using protoplast transformation method (Tunlid et al., 1999; Liu et al., 2020). Hygromycin-resistant transformants were selected on PDAS medium containing 200 mg/ml hygromycin B (Amresco, Solon, United States; Zhen et al., 2019; Liu et al., 2020). Two deletion mutants of *Aolatg4* gene were verified using PCR amplification and Southern blotting analyses as described previously (Xie et al., 2019, 2020).

### Generation of AoAtg8-GFP Fusion Cassette

The promoter (1,345 bp) of the *Aoatg8* gene in *A. oligospora* was amplified with the primers AoAtg8p-f and AoAtg8p-r, and the gene *Aoatg8* was amplified with the primers AoAtg8-f and AoAtg8-r (Supplementary Table S1; Ding et al., 2018). The *Aoatg8* gene was integrated into the *Bsr*GΙ sites of the Ppk2 vector, and the corresponding promoter sequence was inserted into *Xho*Ι; the GFP gene was cloned at the *Bsr*GΙ and *Xho*Ι sites. The plasmid ppk-GFP-atg8 was transformed into the WT and Δ*Aolatg4* mutant strains, as described previously (Liu et al., 2008).

### Analyses of Mycelial Growth Under Different Media and Stress Conditions

For vegetative growth, *A. oligospora* WT strain and its derived mutants (Δ*Aolatg4*) were propagated on PDA plate for 5 days at 28°C and then placed onto fresh media (CMY, TG, and PDA) to assess mycelial growth as described previously (Zhen et al., 2018). For stress tolerance analysis, WT and mutants were inoculated on the center of plates with stress conditions (TG containing 0.04, 0.06, and 0.08 mM menadione and Congo red, as well as 0.3, 0.5, and 0.7 M sorbitol), and cultured at 28°C for 7 days (Zhen et al., 2019). Relative growth inhibition (RGI) value of fungal strain was determined as previously described (Chen et al., 2014; Ma et al., 2020). Each experiment was performed three times.

To determine the transcription of genes *Aolatg4* at the different growth stages, the fungus was incubated on CMY at 28°C, and the mycelia were collected at 3, 4, 5, 7, and 9 days, respectively (Zhen et al., 2019). Moreover, the WT and mutant were inoculated on CM medium for 48 h, and then the mycelia were transferred into MM-N and MM-C, as well CM medium containing 0.05 mg/ml rapamycin for 6 h at 28°C, respectively, and mycelial samples were collected to analyze the transcription of gene *Aolatg4* under chemical and nutrient stresses.

### Analysis of Conidia Yields and Transcription of Sporulation-Related Genes

Conidia yields were determined after the fungal colonies were cultivated on CMY plate at 28°C for 7 days. To restore conidiation, the fungal colonies were cultivated on CMY medium supplemented with 10 or 20 mM glucose. The spores of the colonies was scraped harvested in micro-centrifuge tubes (Biosharp Life Sciences, Hefei, China), the conidia yields of the WT and *ΔAolatg4* mutants were calculated as described previously (Deng et al., 2012). To determine the transcription of sporulation-related genes at the different growth stages, the WT and mutant were incubated on CMY at 28°C, and their mycelia were collected at 3, 5, and 7 days, respectively.

### Confocal Microscopy and Transmission Electron Microscopy Assays

Hyphae expressing the fusion gene of *GFP-atg8* were cultured in CM medium at 28°C and 180 rpm for 48 h, and then transferred into MM-N medium for 6 h at 28°C in a 180 rpm shaker (Lv et al., 2017). For detection of the expression of *GFP-Atg8* in trap cells, 50 μl conidial suspensions (10^5^ conidia per ml) of the WT and mutants were inoculated on MM-N plate contained cellophane. After 24-h incubation at 28°C, ~100 nematodes (*C. elegans*) were added to induce trap formation. Finally, the localization of GFP-Atg8 in hyphae and traps was observed using a confocal laser scanning microscope (Leica, Mannheim, Germany). FM4-64 (Invitrogen, Carlsbad, United States) was used for vacuole staining as described previously (Ma et al., 2020). DAPI (Invitrogen, Carlsbad, United States) was used for nuclear staining as described previously (Xie et al., 2020).

### Trap Formation of *A. oligospora* and Bioassay

For analysis of trap formation induced by nematodes, conidia harvested from 7-day-old CMY cultures were resuspended to 1 × 10^5^ conidia per ml in sterile water. A fifty microliter conidial suspension of the WT and mutants was inoculated on separate WA plates and incubated at 28°C for 2 days (Xie et al., 2019). *Arthrobotrys oligospora* mycelia and *ΔAolatg4* mutants were added to ~200 nematodes on each plate. Trap formation was observed at the time intervals, 12, 24, and 36 h under a light microscopy (Olympus, Tokyo, Japan). Staining with 0.1% Calcofluor White (CFW, Sigma-Aldrich, United States) was carried out to observe trap formation induced by nematodes (Xie et al., 2020). Trap production and nematode death rate were quantified as the total number of traps and nematodes present per unit area of the plates in WT and Δ*Aolatg4* mutants.

### Quantitative Real-Time PCR Analysis

Total RNAs from WT and mutants were extracted using a RNA miniprep Kit (Axygen, Jiangsu, China), and reverse-transcribed into cDNAs. The cDNA samples were used as the template to determine the transcription of sporulation-related genes with paired primers (Supplementary Table S2; Xie et al., 2020). Real-Time PCR (RT-PCR) was performed to analyze the transcription of genes as described previously (Yang et al., 2013). *β*-Tubulin gene (*Aotub*) was used as an internal reference and transcript levels were calculated by the 2^−ΔΔCt^ method (Livak and Schmittgen, 2001).

### Statistical Analysis

Data are presented as the mean ± standard deviation (SD). Prism 5 (GraphPad, San Diego, CA, United States) was used for the photographs and statistical analyses (one-way ANOVA). Tukey’s honestly significant difference (HSD), *p* < 0.05, was considered to indicate significant differences (Ma et al., 2020).

## Results

### Sequence and Phylogenetic Analyses of AolAtg4

The gene *Aolatg4* encodes a 444-amino acid polypeptide with a molecular mass of 49.8 kDa and an isoelectric point of 4.8, which contains a conserved peptidase C54 (IPR005078) domain. AolAtg4 shares a high degree of similarity with orthologs from various fungi. It has 89.6 and 93.2% identity with the homologous Atg4 from two species of NT fungi *Duddingtonia flagrans* and *Dactylellina haptotyla*, respectively; 42.7–56.8% sequence identity with the orthologs from other filamentous fungi; and the lowest identity (32.3%) with its *S. cerevisiae* ortholog. Atg4 orthologs from diverse fungi was divided into two clades (A and B); Atg4 orthologs from three species of NT fungi clustered into clade B, and Atg4 from other filamentous fungi clustered into clade A (Supplementary Figure S1).

### AolAtg4 Regulates Mycelial Growth, Cell Nuclei, and Septal Development

**Figure 1:**
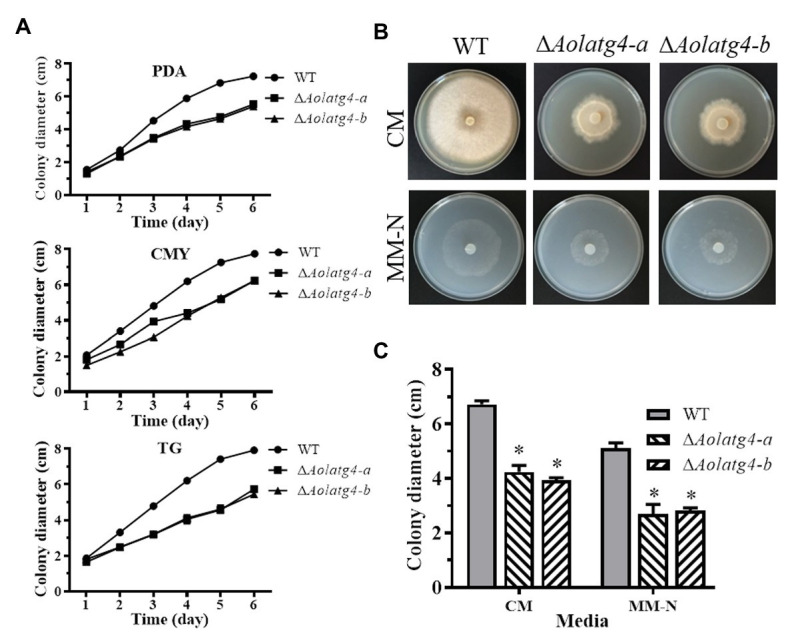
Comparison of mycelial growth of the wild type (WT) and *ΔAolatg4* mutants on different meida. **(A)** Colony diameters of fungal strains incubated on potato dextrose agar (PDA), CMY, and tryptone glucose (TG) media. Each experiment was performed three times. **(B)** Colony morphology of fungal stains incubated on CM and MM-N media at 28°C for 7 days. **(C)** Colony diameters of fungal strains incubated on CM and MM-N plates for 7 days. Error bars: standard deviation, asterisk: significant difference between mutant and WT (Tukey’s HSD, *p* < 0.05).

Two ∆*Aolatg4* mutants (a and b) were verified using PCR amplification and Southern blot methods (Supplementary Figure S2). Mycelial growth showed a statistically significant difference between the WT strain and Δ*Aolatg4* mutants on different media (CMY, TYGA, and TG; Figure 1A and Supplementary Figure S3). The colony diameter of the WT and ∆*Aolatg4* mutants was 7.75 ± 0.23 and 6.24 ± 0.12 cm, respectively on CMY plate in 6 days (Figure 1A). In TG, the colony diameter of the ∆*Aolatg4* mutants was 5.45 ± 0.13 cm after incubation for 6 days, which was significantly smaller than the 7.92 ± 0.16 cm colony diameter of the WT strain (Figure 1A). In addition, the Δ*Aolatg4* mutants also showed different hyphal growth rate compared to the WT strain on CM and MM-N. The Δ*Aolatg4* mutants displayed decreased growth and sparse aerial hyphae on CM medium compared to the WT strain. The Δ*Aolatg4* mutants also grew slower than the WT strain on MM-N (Figures 1B,C).

The transcription of gene *Aolatg4* was increased in *A. oligospora* during the conidiation stage (7–9 days; Supplementary Figure S4A). Moreover, the transcript level of gene *Aolatg4* was upregulated under chemical and nutrient stresses, including MM-N, MM-C, and CM containing 0.05 mg/ml rapamycin (Supplementary Figure S4B).

The cell nuclei were observed in WT and Δ*Aolatg4* mutants using DAPI staining. The hyphal cells of the WT contained 11–22 nuclei, whereas only 5–9 nuclei were observed in Δ*Aolatg4* mutants (Supplementary Figure S5A). Moreover, more septa were observed in the hyphae of the Δ*Aoatg4* mutants than in the WT strain using CFW staining (Supplementary Figure S5B).

### AolAtg4 Plays a Critical Role in Conidiation

**Figure 2:**
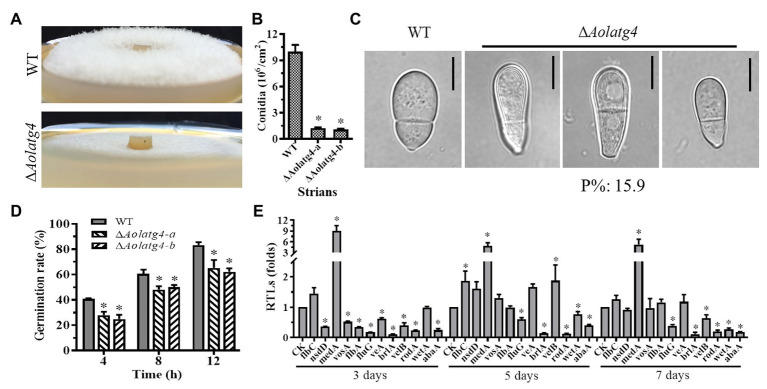
Comparison of aerial hyphae, conidiation, and the transcript of sporulation-related genes between the WT and Δ*Aolatg4* mutants. **(A)** Comparison of aerial hyphae between the WT and Δ*Aolatg4* mutants; the fungal strains were incubated on CMY for 7 days at 28°C. **(B)** The conidia yields collected from strains grown in CMY for 7 days. **(C)** The conidia of the WT and Δ*Aolatg4* mutants. P%: the percentage of the deformed spores in the mutant. **(D)** Spores of fungal strains germinate; the spores of the WT and Δ*Aolatg4* mutants were incubated in MM-N liquid medium for 4, 8, and 12 h. **(E)** Comparison of sporulation-related genes between the WT and Δ*Aolatg4* mutants. CK was denoted as a standard for statistical analysis of the RTL. Each experiment was performed three times. Error bars: standard deviation, asterisk: significant difference between mutant and WT (Tukey’s HSD, *p* < 0.05).

The wild type strain had luxuriant aerial hyphae when cultured on CMY plates, in contrast to the sparse aerial hyphae in the ∆*Aolatg4* mutants (Figure 2A). Spore production in the ∆*Aolatg4* mutants was significantly decreased. The mutants produced (1.2 ± 0.08) × 10^6^ conidia/cm^2^, whereas the WT strain produced (10 ± 0.24) × 10^6^ conidia/cm^2^; the conidial yield of the mutants was decreased by 88.4% (Figure 2B). Meanwhile, the conidiation of the ∆*Aolatg4* mutant was partially restored when supplemented with 10 or 20 mM glucose (Supplementary Figure S6). Furtherly, we determined conidial germination on MM medium, at 4 h, 27.9% of the conidia of ∆*Aolatg4* mutants germinated, compared to 40.8% of those of the WT strain, and 12 h later, the spore germination rates of the mutants and WT strain were 62.2 and 83.3%, respectively (Figure 2D). Moreover, 15.9% of the spores of the mutants were deformed compared to the WT strain (Figure 2C).

The transcript levels of 12 sporulation-related genes changed in the ∆*Aolatg4* mutant compared to those of the WT strain. Among them, nine genes (*abaA*, *brlA*, *flbA*, *fluG*, *flbC*, *nsdD*, *rodA*, *veA*, and *vosA*) were remarkably downregulated on day 3, and six genes including *abaA*, *fluG*, *brlA*, *rodA*, *velB*, and *wetA* were all downregulated on day 3, 5, and 7. However, *medA* was remarkably upregulated at three time points (Figure 2E).

### AolAtg4 Regulates Stress Resistance in *A. oligospora*

**Figure 3:**
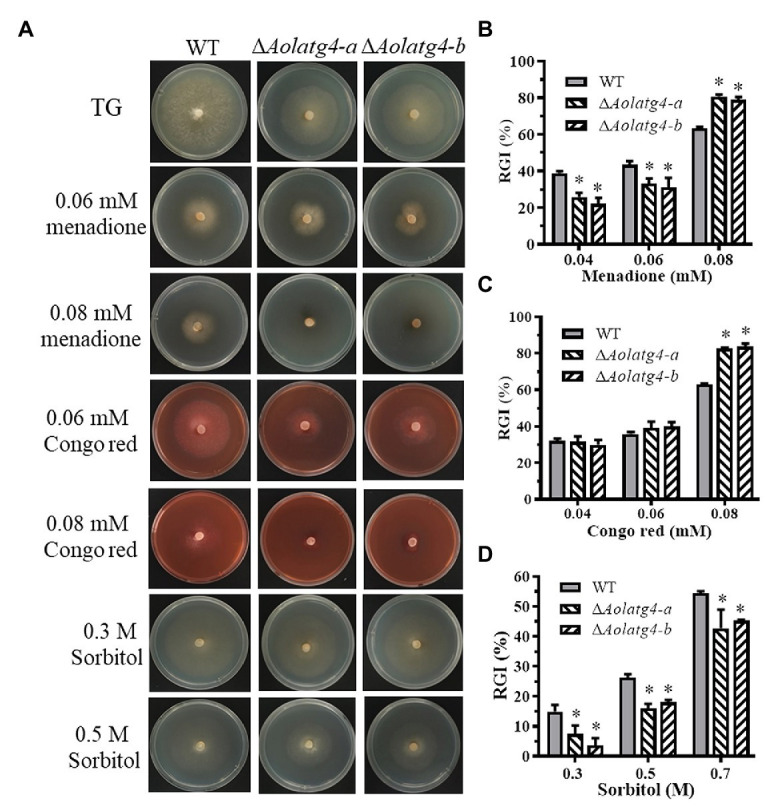
Comparison of stress resistance between WT and Δ*Aolatg4* mutants. **(A)** The WT strain and Δ*Aolatg4* mutant were incubated on TG medium containing menadione, sorbitol, and Congo red. **(B)** Relative growth inhibition (RGI) values of the fungal strains incubated on TG medium containing 0.04–0.08 mM menadione for 7 days. **(C)** RGI values of the fungal strains incubated on TG medium containing 0.04–0.08 mM Congo red for 7 days. **(D)** RGI values of the fungal strains incubated on TG medium containing 0.3–0.7 M sorbitol for 7 days. Each experiment was performed three times. Error bars: standard deviation, asterisk: significant difference between mutant and WT (Tukey’s HSD, *p* < 0.05).

The mycelial growth of WT was affected by TG plate-supplemented chemical stress (menadione, Congo red, and sorbitol), whereas the growth of the mutants was promoted by 0.04–0.06 mM and inhibited by 0.08 mM menadione; the growth of the mutants showed no change with 0.04–0.06 mM Congo red but was inhibited by 0.08 mM Congo red; in contrast, the growth of the mutants was promoted by 0.3–0.7 M sorbitol (Figure 3A). The RGI value of the ∆*Aolatg4* mutants (83%) was higher than that of the WT strain (63%) on TG plates containing 0.08 mM Congo red or menadione (Figures 3B,C), whereas the RGI values of the mutant (5.6, 17.0, and 44%) were lower than that of the WT (14.9, 26.3, and 54.3%) on TG plates containing 0.3, 0.5, and 0.7 M sorbitol (Figure 3D), respectively.

### AolAtg4 Regulates Autophagic Process

**Figure 4:**
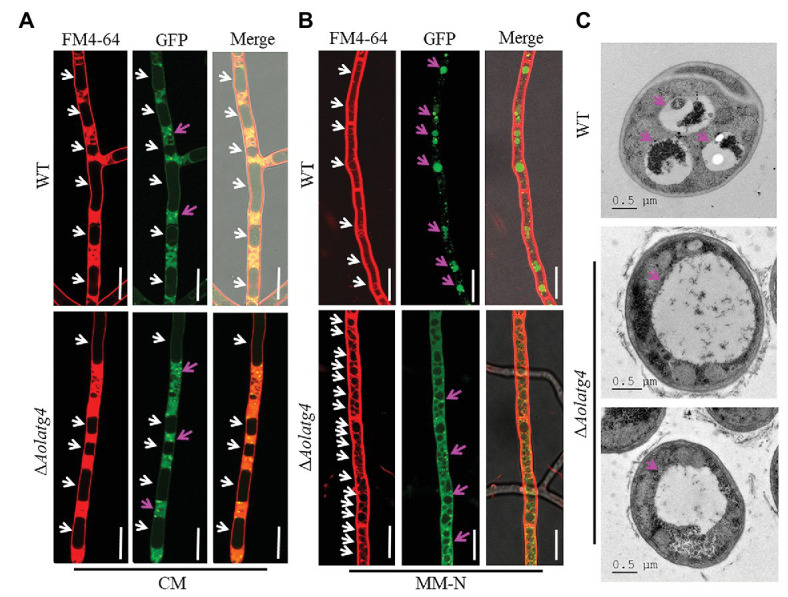
Observation of autophagosomes in the hyphal vacuoles of the WT and ∆*Aolatg4* mutants. **(A)** Observation of the WT and ∆*Aolatg4* mutants expressing GFP-Atg8; the strains were cultivated in liquid CM medium at 28°C for 48 h. **(B)** Observation of the WT and ∆*Aolatg4* mutants expressing GFP-Atg8; the strains were grown in liquid CM medium at 28°C for 24 h, and then transferred to liquid MM-N medium for 6 h. The vacuoles were stained by FM4-64 and examined by fluorescence microscopy. White arrow: vacuoles; red arrow: autophagosomes. Scale bars = 10 μm. **(C)** The vacuoles of hyphal cells were observed using transmission electron microscopy. Arrows indicate the vacuole. Scale bars = 0.5 μm.

**Figure 5:**
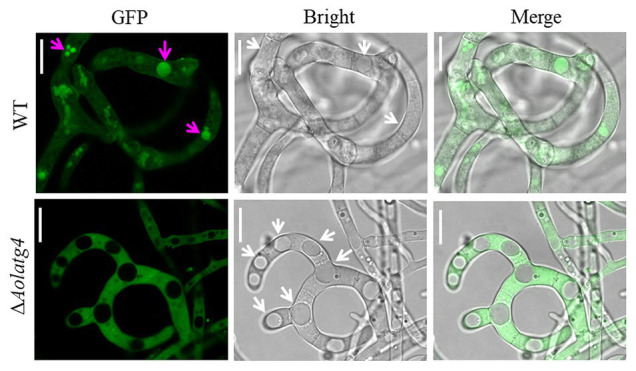
Detection of autophagosomes in the traps of the WT and ∆*Aolatg4* mutants. Conidial suspensions of the indicated strains were inoculated on MM-N plate contained cellophane. After 24-h incubation at 28°C, about 100 nematodes were added on each plate. Autophagosomes in traps were observed using fluorescence microscopy. White arrow: vacuoles; red arrow: autophagosomes. Scale bars = 10 μm.

To determine the blockage of the autophagic pathway in the ∆*Aolatg4* mutants, we constructed the GFP-Atg8 fusion protein and expressed it in the WT and ∆*Aolatg4* mutants. These strains were cultured in CM medium (rich-nutrient condition) for 48 h, few autophagosomes were observed in peripheral of vacuole in the hyphae of the WT and ∆*Aolatg4* mutants, whereas no GFP signal was accumulated in vacuoles (Figure 4A). When fungal strains were cultured in CM medium for 24 h, and then transferred to MM-N medium (nitrogen starvation condition) and incubated for 6 h. Under starvation stress, the GFP signals were migrated into the mycelial vacuoles of the WT strain, whereas no GFP signal was accumulated in vacuoles of the ∆*Aolatg4* mutants but instead was distributed dispersedly in hyphal cells (Figure 4B). Moreover, the vacuoles were significantly increased in numbers in the mutants compared to the WT strain (Figure 4B). To confirm whether autophagy was affected by deletion of the gene *Aolatg4* in *A. oligospora*, the autophagosomes in vacuoles were observed in the WT and ∆*Aolatg4* mutants using TEM. When fungal strains were cultured in MM-N medium for 6 h, autophagosomes were clearly observed in the vacuoles of the WT strain, while few autophagosomes or autophagosome-like structures (22.6% vs. to the WT strain) were observed in vacuoles of ∆*Aolatg4* mutants (Figure 4C). Moreover, we further determine the localization of GFP-Atg8 in traps of the WT and mutants. GFP signals accumulated in vacuoles of the traps and adjacent cells of the WT strain, and no GFP signal was observed in vacuoles of the traps in the ∆*Aolatg4* mutants (Figure 5).

### AolAtg4 Regulates Trap Formation and Pathogenicity

**Figure 6:**
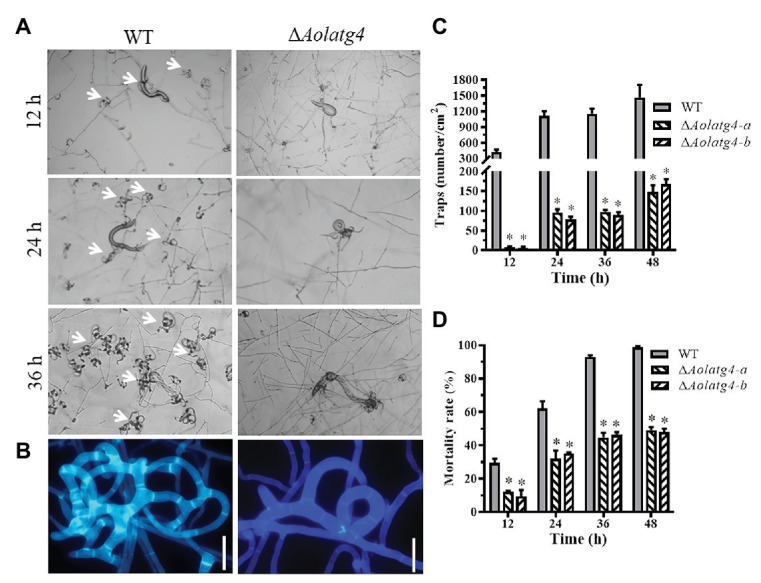
Analysis of trap formation and nematicidal activity of the WT and Δ*Aolatg4* mutants. **(A)** Traps induced by nematodes in WT and mutants at 12, 24, and 36 h. White arrow: traps. **(B)** The enlarged traps produced by the WT and mutants at 48 h; the traps were stained with Calcofluor White (CFW; 10 μg/ml). Scale bars = 10 μm. **(C)** The traps produced by the WT and mutants at different time points. **(D)** The mortality rate (%) of nematodes at different time points. Each experiment was performed three times. Error bars: standard deviation, asterisk: significant difference between mutant and WT (Tukey’s HSD, *p* < 0.05).

The vegetative hyphae of *A. oligospora* and other NT fungi can develop diverse traps for nematode predation. After addition of nematodes for 12 h, fresh traps containing one or two hyphal loops (immature traps) were observed on the plates culturing the WT strain, whereas few traps were observed on the plates culturing the ∆*Aolatg4* mutants. Mature traps containing multiple hyphal loops were observed at 24 and 36 h in the WT strain (Figures 6A,B); 812 and 1,150 traps were observed in the WT strain at 24 and 36 h, respectively, whereas 96 and 98 traps per plate were produced by the ∆*Aolatg4* mutants at 24 and 36 h, respectively (Figure 6C). We further observed the traps produced by the WT and ∆*Aolatg4* mutants; the traps of the WT strain consisted of six or more hyphal loops, whereas traps developed by the ∆*Aolatg4* mutants contained only 2–3 hyphal loops (Figure 6B) even at 48 h or longer.

The fungal strains began to capture nematodes with trap formation; 29.6% of nematodes were captured by the WT strain and 10.8% of nematodes were captured by the ∆*Aolatg4* mutants at 12 h. Subsequently, 62.2, 92.8, and 98.6% nematodes were captured by the WT strain at 24, 36, and 48 h (Figure 6D), respectively. In contrast, 33.5, 45.5, and 48.5% of nematodes were captured by the Δ*Aolatg4* mutants at the corresponding time points (Figure 6D).

## Discussion

Autophagy is a conserved biological process in eukaryotes and contributes to maintaining cellular homeostasis, which is strictly regulated by Atg proteins (Mizushima et al., 2011; Maruyama and Noda, 2018). Recent years, increasing studies have shown that Atg proteins involve in the regulation of vegetative growth, conidiation, infection structure, and virulence of fungi (Liu et al., 2012; Ying and Feng, 2019). Previous research suggested that *Aoatg8* gene is necessary for hyphal growth, sporulation, and trap formation in *A. oligospora* (Chen et al., 2013). In this study, we characterized the gene *Aolatg4* in *A. oligospora*, and our analysis showed that deletion of *Aolatg4* impaired autophagic process, thus affecting diverse phenotypic traits, such as hyphal growth, conidiation, and nematicidal activity in *A. oligospora*.

Deletion of the gene *Aolatg4* caused defective growth on the PDA, CMY, and TG media compared to the WT strain, and mycelial growth of the mutants was also reduced on CM and MM-N media (Figures 1B,C). Meanwhile, the aerial mycelia of the ∆*Aolatg4* mutants became very sparse (Figure 2A). Similarly, deletion of the gene *Moatg4* in *M. oryzae* caused a significant reduction in hyphal growth (Liu et al., 2010), and colonies of the ∆*Fgatg4* mutants were significantly smaller than those of the WT strain under nutrient-rich conditions (PDA plates) in *Fusarium graminearum* (Lv et al., 2017). These analyses show that *atg4* plays pivotal roles in mycelium growth under routine or nitrogen starvation conditions.

Other than mycelial growth, conidiation was also seriously affected in the *ΔAolatg4* mutants. The spore yield of the *ΔAolatg4* mutants was decreased by 88.4%. Further, 15.4% conidia of the mutants became spindly from obovoid, as seen in the WT strain (Figure 2B). The transcript of six sporulation-related genes, such as *abaA*, *brlA*, *fluG*, *rodA*, and *wetA*, was significantly downregulated at the conidiation stage, whereas the *medA* gene was remarkably upregulated at all time points in the Δ*Aolatg4* mutant compared to the WT strain. These genes play a crucial role in conidiation in filamentous fungi. For example, *abaA*, *brlA*, and *wetA* are the key genes encoding central regulators of conidiation in species of *Aspergillus* (Park and Yu, 2012), *F. graminearum* (Son et al., 2013), and *B. bassiana* (Zhang et al., 2019). The *rodA* gene is required for the rodlet layer formation and hydrophobicity in conidia of *A. fumigatus* (Park and Yu, 2016); and gene *velB* is required for conidiation in *A. oligospora*; Δ*AovelB* mutants displayed serious sporulation defects (Zhang et al., 2019). Interestingly, the conidiation defect in the Δ*Aolatg4* mutants could be restored by glucose, suggested that it was directly related to autophagy defect. Similarly, deletion of *Moatg4* caused a significant defective in conidiation and conidial germination in *M. oryzae* (Liu et al., 2010). Moreover, the gene *Moatg8* was significantly induced during asexual development in *M. oryzae*, and the conidial yield was dramatically reduced in the Δ*Moatg4* mutant (Deng et al., 2009). Therefore, *atg4* plays a conserved and pivotal role in conidiation in *A. oligospora* and *M. oryzae*.

Moreover, deletion of the gene *Aolatg4* resulted in an increase in sensitivity to chemical stressors, such as menadione and Congo red (Figure 3). Similarly, deletion of *Hoatg5* caused defective in hyphal growth on CM plates containing cell wall-perturbing agents, including CFW, SDS, and Congo red, in the endophytic fungus *Harpophora oryzae* (Liu et al., 2016). Moreover, the vegetative growth of Δ*Bbatg1* and Δ*Bbatg8* mutants was inhibited by menadione in *B. bassiana* (Ying et al., 2016). These results indicate that autophagy is involved in the regulation of fungal sensitivity to cell wall-disturbing agents and antioxidants.

The ∆*atg4* mutants were almost completely blockage in autophagy and produced few small autophagosomes in *S. cerevisiae* under starvation conditions (Hirata et al., 2017). In filamentous fungi such as *Sordaria macrospora* and *M. oryzae*, orthologs of the *atg4* gene have been characterized in autophagy. In *S. macrospora*, *Smatg4* is required for nonselective macroautophagy and selective macropexophagy (Voigt and Pöggeler, 2013). No autophagosomes were accumulated in the cytoplasm or autophagic bodies in the lumen of vacuoles in Δ*Moatg4* mutants (Liu et al., 2010). Moreover, the Δ*Aoatg4* mutant in *Aspergillus oryzae* indicated a defect in autophagy according to observation of the behavior of GFP-AoAtg8 (Kikuma et al., 2006). In this study, we found that autophagosomes were obviously decreased in the ∆*Aolatg4* mutants under starvation conditions by TEM observation; we further confirmed using the GFP-Atg8 fusion protein that autophagic process was impaired in the hyphae and traps of Δ*Aolatg4* mutant. Our results showed that autophagosome formation was impaired in ∆*Aolatg4* mutants. Therefore, Atg4 plays a vital role in autophagosome formation and autophagic processes in yeast and filamentous fungi.

*Arthrobotrys oligospora* is a common species of NT fungi, and trap formation is an important indicator for the lifestyle switch of NT fungi (Su et al., 2017). In previous study, deletion of the *Aoatg8* gene suppressed nematode-induced autophagy and trap formation, suggested that autophagy plays an important role in trap formation of *A. oligospora* (Chen et al., 2013). In this study, we further characterized the role of *Aolatg4* in trap formation and pathogenicity. Our results showed that *Aolatg4* also contributes to *A. oligospora* autophagy; meanwhile, trap formation and nematicidal activity was remarkably decreased in the ∆*Aolatg4* mutants, and the mutant only produced immature traps containing 2–3 hyphal loops. Similarly, deletion of the gene *Moatg4* causes loss of appressorium ability to penetrate rice and barley (Liu et al., 2010). These results showed that *atg4* regulates the development of infection structure, thus playing a vital role in pathogenicity in pathogenic fungi.

In summary, our results demonstrate that AolAtg4 plays an important role in hyphal growth, sporulation, stress resistance, trap formation, and regulation of autophagic process in *A. oligospora*. Our results provide a basis for investigating the roles of *atg* genes in *A. oligospora* and other NT fungi, which will help to explore the regulation mechanisms of vegetative growth, development, and differentiation of NT fungi.

## Conclusion

We identified and characterized an autophagy gene, *Aolatg4*, from the fungus *A. oligospora*. *Aolatg4* plays a crucial role in autophagic process, and is important for conidiation, trap formation, and resistance to oxidants and cell-wall-perturbing agents in this fungus. Except for above phenotypic traits, *Aolatg4* also regulates the development of cell nuclei and mycelial septa in *A. oligospora*. Our findings provide a novel understanding of *atg* genes that regulate hyphal growth, conidiation, trap formation, and pathogenicity of NT fungi.

## Data Availability Statement

The original contributions presented in the study are included in the article/Supplementary Material, and further inquiries can be directed to the corresponding author.

## Author Contributions

JY conceived and designed the study. DZ and JY wrote the manuscript. DZ conducted the experiments. MX, NB, and LY analyzed the data. JY and K-QZ revised the manuscript. All authors contributed to the article and approved the submitted version.

### Conflict of Interest

The authors declare that the research was conducted in the absence of any commercial or financial relationships that could be construed as a potential conflict of interest.

## Footnotes

^1^http://www.ebi.ac.uk/interpro/
