# Supplementary material for: The Autophagy-Related Gene Aolatg4 Regulates Hyphal Growth, Sporulation, Autophagosome Formation, and Pathogenicity in Arthrobotrys oligospora
Source: Front Microbiol. 2020 Nov 16;11:592524. doi: 10.3389/fmicb.2020.592524 (PMC7701090; doi:10.3389/fmicb.2020.592524)
Supplement: Supplementary file 1 [file Data_Sheet_1.pdf]

Supplementary materials

1. Supplementary Figures

Supplementary Figure S1. A neighbor-joining phylogenetic tree of the sequences orthologous to Atg4 from different fungi. GenBank accession numbers are provided in brackets. The Atg4 of *Saccharomyces cerevisiae* (NP_014176) is used as an outgroup.

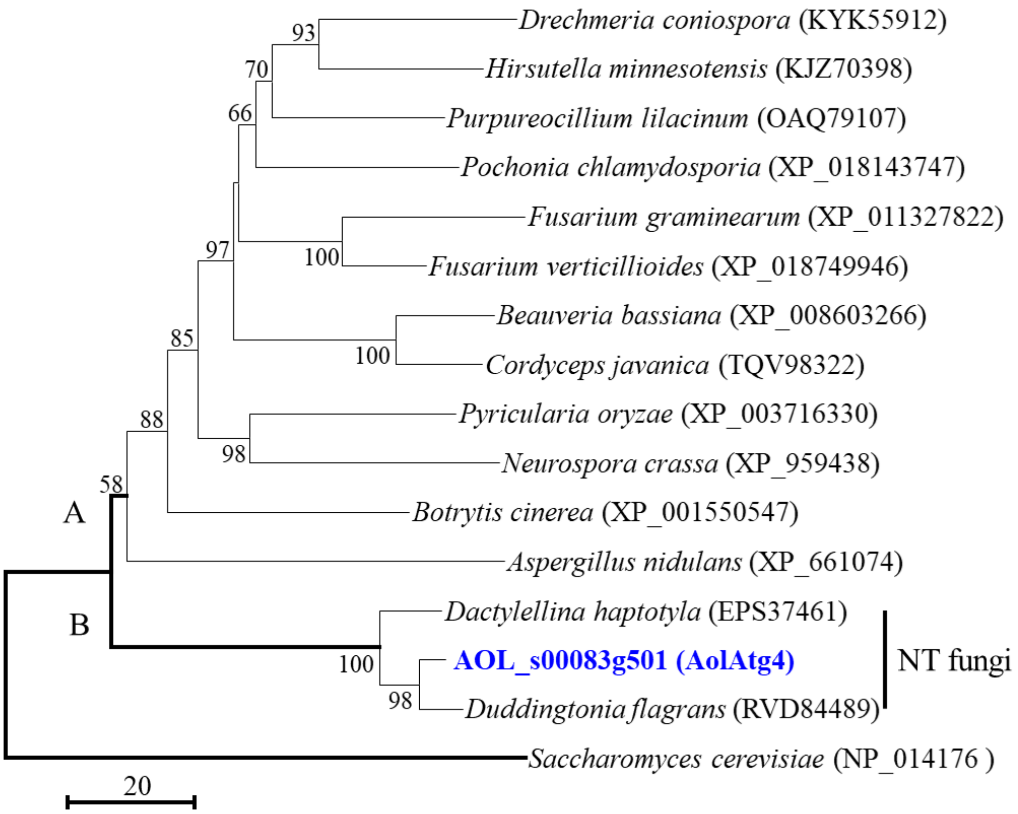

Supplementary Figure S2. Knock-out and verification of the gene *Aolatg4* in *A. oligospora*. (A) Diagrammatic sketch of homologous recombination of *Aolatg4*. The homologous flanking sequences of the target gene, Southern blot probe, and the *Sna*BI restriction enzyme sites are marked. P, probe. **(B)** Positive transformants were verified by PCR amplification. M, DNA ladder. WT, the wild-type strain; M1 and M2, the *Aolatg4* gene deletion strains. **C.** The Δ*Aolatg4* mutants were confirmed by blotting analysis.

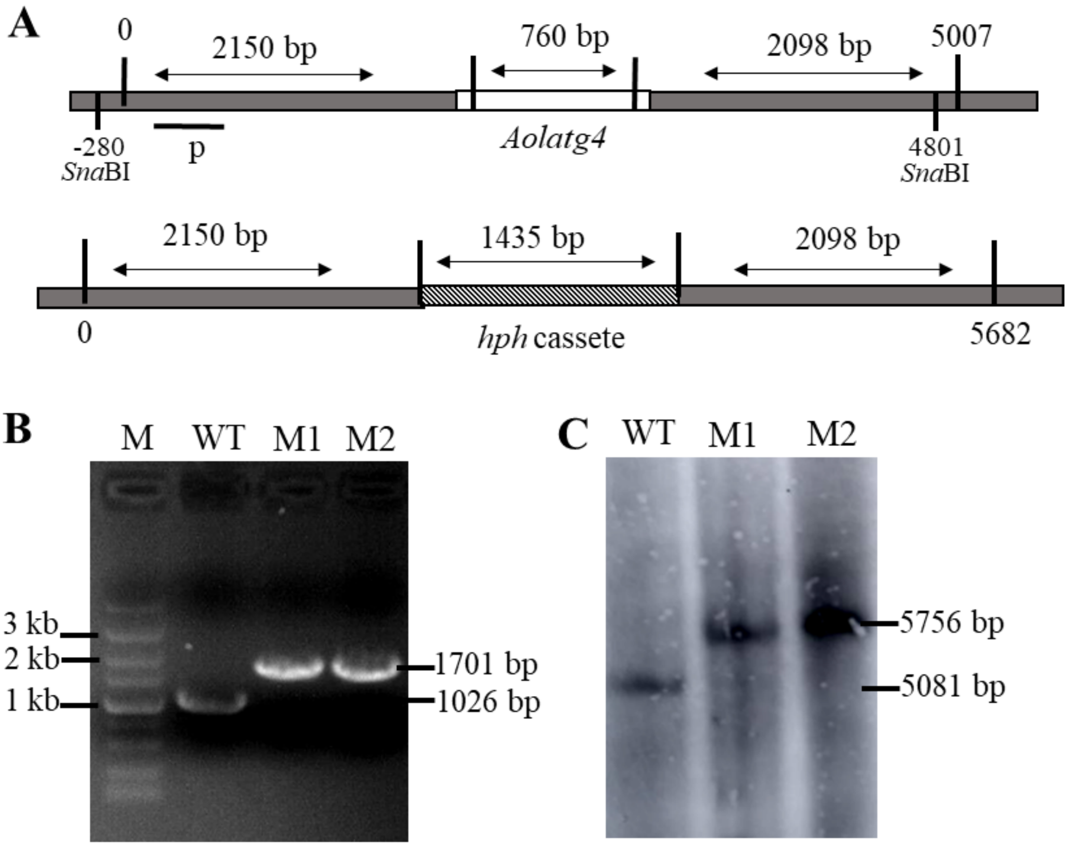

Supplementary Figure S3. Colonies from the WT and Δ*Aolatg4* mutants were incubated on PDA, CMY, and TG plates for 6 days at 28 ℃.

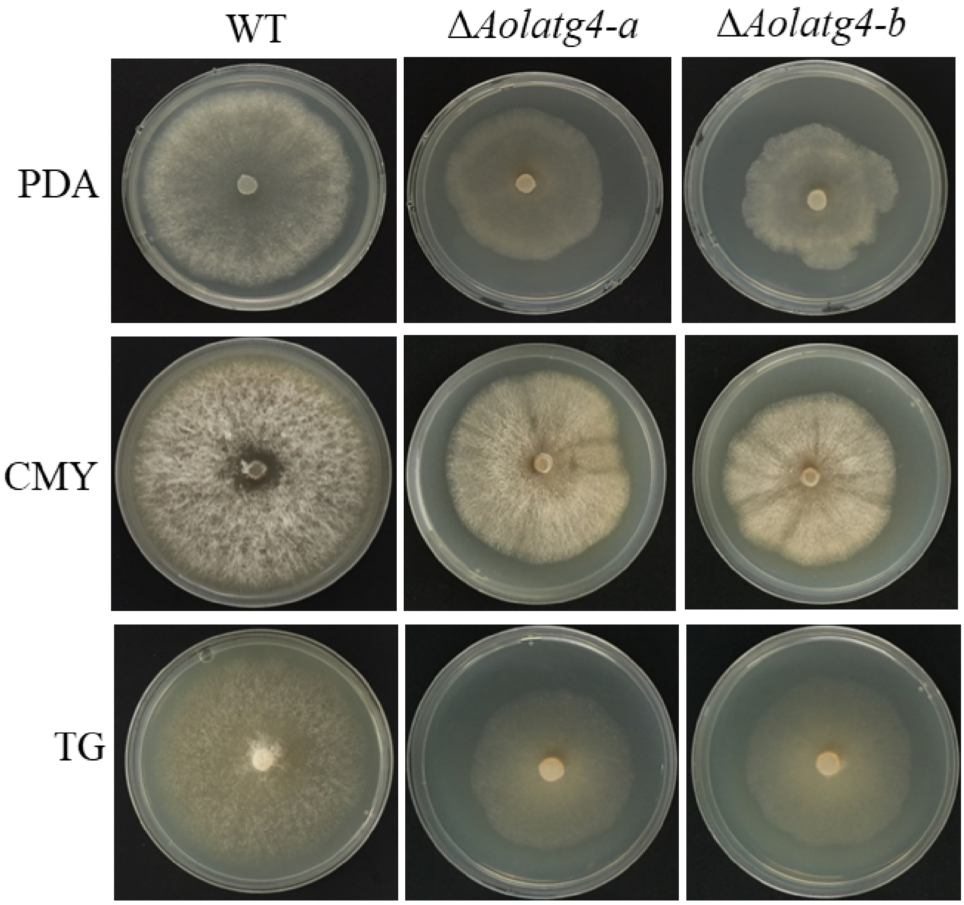

Supplementary Figure S4. Transcript levels of gene *Aolatg4* in *A. oligospora* at different growth stages and stressed media. A. The relative transcription levels (RTLs) of gene *Aolatg4* at different growth stages upon incubation on CMY for 3, 4, 5, 7, and 9 days. Error bars: SD from three replicates, asterisk: significant difference between 4 and 9 d and that at 3 d (Tukey's HSD, $p < 0.05$). B. RTLs of gene *Aolatg4* under chemical and nutrient stresses, including MM-N, MM-C, and CM containing 0.05 mg/ml rapamycin. Error bars: SD from three replicates, asterisk: significant difference between stressed media and CM medium (Tukey's HSD, $p < 0.05$).

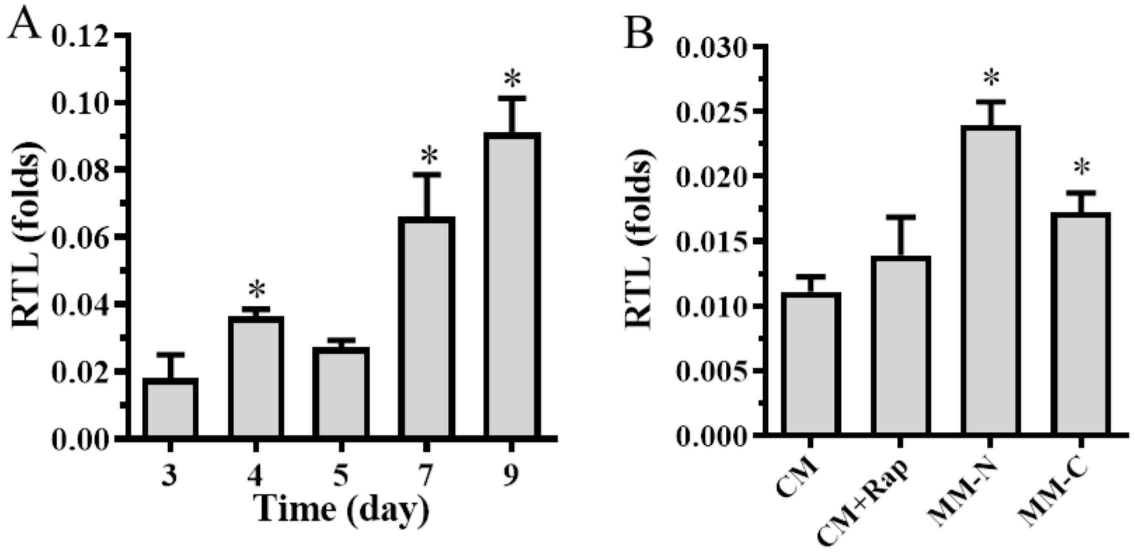

Supplementary Figure S5. Comparison of the cell nucleus and septum between the WT and mutant strains. A. Observation of cell nucleus and septum between the WT and mutant strains. White arrow, cell nucleus; red arrow, septum. B. Number of cell nuclei in the WT and mutant. C. Observation of hyphal septum in the WT and mutant. White arrow, septum. Error bars: SD from three replicates, asterisk: significant difference between mutant and WT (Tukey's HSD, $p < 0.05$).

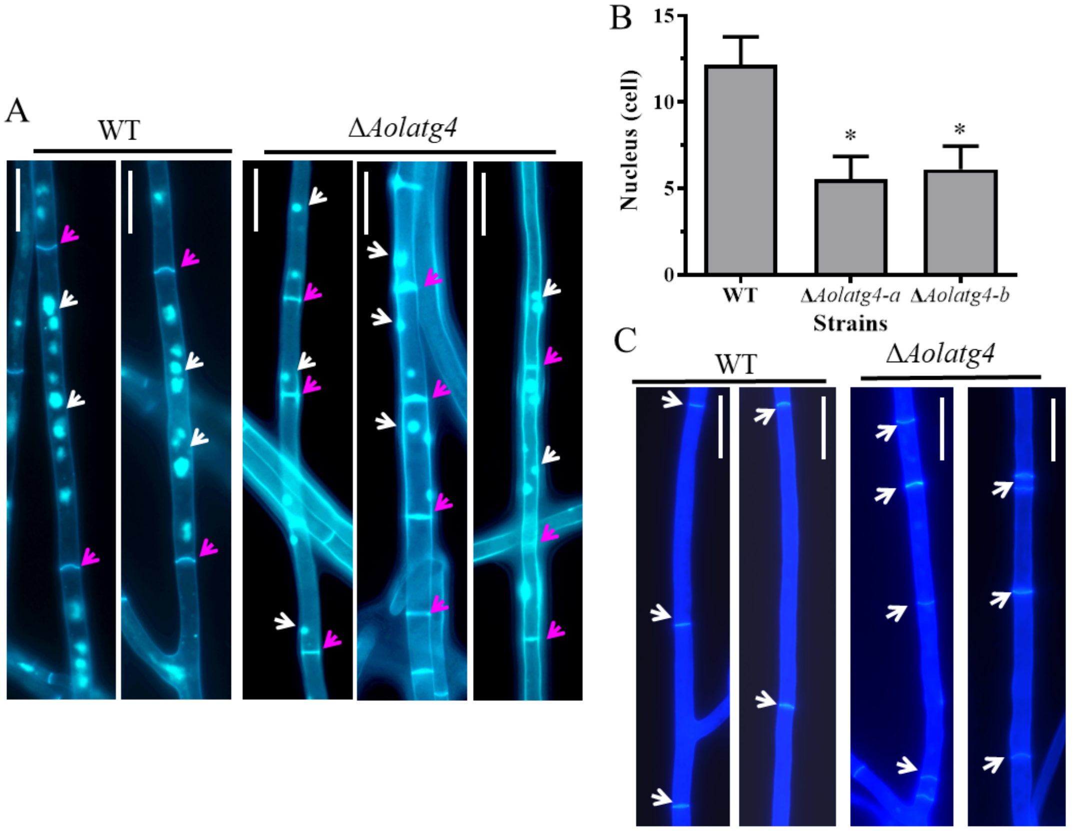

Supplementary Figure S6. Comparison of conidiation of the WT and Δ*Aolatg4* mutant strains on CMY and CMY supplemented with 10 or 20 mM glucose. Error bars: SD from three replicates.

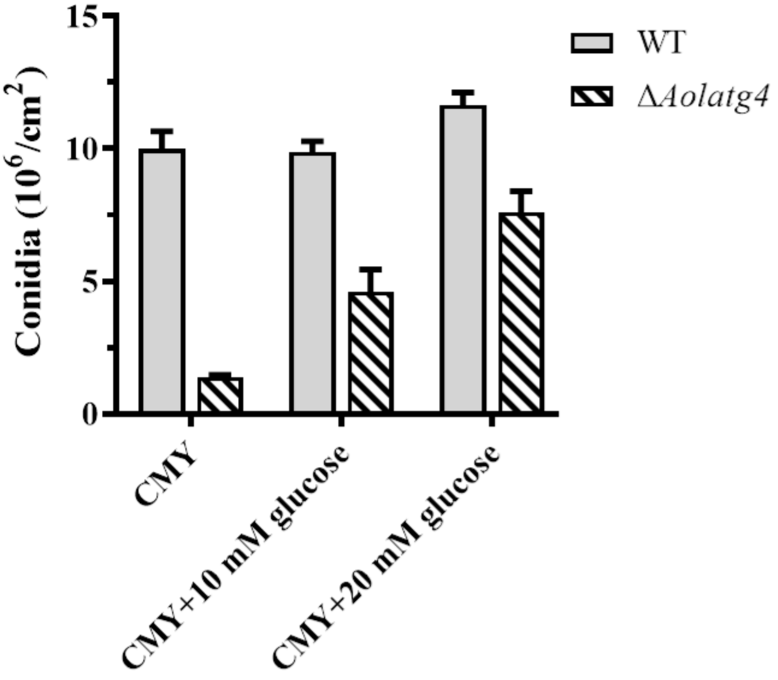

2. Supplementary tables

Supplementary Table S1. List of primers used in this study.

Primers	Sequence (5′-3′)	Description
AolAtg4-5f	GTAACGCCAGGGTTTTCCCAGTCACGACGTTCAGTTCACTCCCCAAG	Amplify the *Aolatg4* gene
AolAtg4-5r	ATCCACTTAACGTTACTGAAATCTCCAACTCTCGTGGGTATAAGGTTAG	5’ flank
AolAtg4-3f	CTCCTTCAATATCATCTTCTGTCTCCGACCGGCAGATGTTGAGGAT	Amplify the *Aolatg4* gene
AolAtg4-3r	GCGGATAACAATTTCACACAGGAAACAGCGCATCAGAATCTTCGTGTC	3’ flank
Hph-f	GTCGGAGACAGAAGATGATATTGAAGGAGC	Amplify the *hph* cassette
Hph-r	GTTGGAGATTTCAGTAACGTTAAGTGGAT	
YZatg4-5f	ATCCCGACTGACTGTGG	Verify the transformants
YZatg4-3r	ACCCGCCTGTCAAGAAT	
probe5f	TTACTGAAGACCACGAAGAACA	Make Southern blotting
probe3r	TTAGCGGAATGTTAAATGGG	probe
AolAtg8p-f	AGGAGGTCAAAGCTTACCAGATACGATTAATCCAGCATCTCACGACC	Amplify the promoter
AolAtg8p-r	ATGGTGAGCAAGGGCGAGGAGCTTCATAGATTTGGGTGTCGGTT	fragment of *Aolatg8*
AolAtg8-f	GTACAGCTCGTCCATGCCGAGAGTGAATGGCACGATCAAAGTT	Amplify the gene *Aolatg8*
AolAtg8-r	AAGTAAACAATCAATCCATTTCGCTATTCAGGCAAGGTAC	

Supplementary Table S2. Paired primers of sporulation-related genes used for RT-PCR analysis.

**Sporulation genes**	Sequence (5′-3′)	**Sporulation genes**	Sequence (5′-3′)
AOL_s00169g18	18-5F-AAGCTACACCCAATCAACGC	AOL_s00054g700	700-5F-CAAACCACCCACCACCAAAT
(*veA*)	18-3R-TTGCGATGCTGACGATCTTG	(*vosA*)	700-3R-GGATGGACAGGAGAAGGACC
AOL_s00007g157	157-5F-CTCTCCGGCAAAGACAATCG	AOL_s00215g516	516-5F-TTCAAACGCAGCTCCTTCAC
(*flbC*)	157-3R-GTCGACTGAGGATAGTAGCT	(*flbA*)	516-5R-AAGCGGGTTGACAGATGAGA
AOL_s00075g211	211-5F-ATTACGGCCGCCTAGTAGTC	AOL_s00210g120	120-5F-TCCGGCCCAATGATTCAGAA
(*nsdD*)	211-3R-CTCGTTTGGACCTGGTTGTG	(*medA*)	120-5R-AGATCGCAGGAACATGGTGA
AOL_s00043g361	361-5F-GATTCCAGTCCCGTGAATTC	AOL_s00097g514	514-5F-AACTCCATCACCATCCGTAA
(*fluG*)	361-3R-GCTAAGGAGAGGATGGGCAT	(*brlA*)	514-5R-CAGGATATTCGGCACTCA
AOL_s00006g570	570-5F-GCGGATCCAACATGAAGCTT	AOL_s00173g221	221-5F-CCCTGTGCTACTATTGCTAC
(*rodA*)	570-3R-GGTTGACAACTGGGATGCTG	(*wetA*)	221-5R-CCGTTGCGAGCATTTCTT
AOL_s00080g63	63-5F-AACTTTATGCGCCTTGTCGT	AOL_s00054g811	811-5F-ATTCCGCAACTTCTCCCTCA
(*abaA*)	63-3R-TTGGCTAGGTGGTCTGTACG	(*velB*)	811-3R-GGCATGTTTGGATTCTGGGG

**β-tubulin gene**	Sequence (5′-3′)
AOL_s00076g640	tubA-F-CCACCTTCGTCGGTAACTC
(*Aotub*)	tubA-R-TCGTCCATACCCTCACCAG
